# Rapid screening of SARS-CoV-2 variants, a key tool for pandemic surveillance

**DOI:** 10.1038/s41598-023-37866-8

**Published:** 2023-07-08

**Authors:** Irene Muñoz-Gallego, María Ángeles Meléndez Carmona, Carmen Martín Higuera, Esther Viedma, Rafael Delgado, María Dolores Folgueira

**Affiliations:** 1 Laboratory of Virology, Microbiology Department, Hospital Universitario, 12 de Octubre, Avda de Córdoba s/n, 28041 Madrid, Spain; 2 Biomedical Research Institute imas12, Hospital Universitario 12 de Octubre, Madrid, Spain; 3 Department of Medicine, School of Medicine, Universitario Complutense, Madrid, Spain

**Keywords:** Molecular biology, Microbiology, Virology

## Abstract

The utility of reverse transcription-polymerase chain reaction (RT-PCR) in analysis SARS-COV-2 variants was evaluated. RT-PCR tests were used to analyse the majority of new SARS-CoV-2 cases (n = 9315) in a tertiary hospital (Madrid, Spain) throughout 2021. Subsequently, whole genome sequencing (WGS) was conducted on 10.8% of these samples (n = 1002). Notably, the Delta and Omicron variants emerged rapidly. There were no discrepancies between RT-PCR and WGS results. Continuous surveillance of SARS-CoV-2 variants is essential, and RT-PCR is a highly useful method, specially during periods of high COVID-19 incidence. This feasible technique can be implemented in all SARS-CoV-2 laboratories. However, WGS remains the gold standard method for comprehensive detection of all existing SARS-CoV-2 variants.

## Introduction

Severe acute respiratory syndrome coronavirus 2 (SARS-CoV-2), which causes coronavirus disease 2019 (COVID-19), emerged in China in late 2019 and has resulted in the ongoing worldwide pandemic^1^.

Coronaviruses encode four major structural proteins: spike (S), membrane (M), envelope (E), and nucleocapsid (N). The S protein is required for entry of the infectious virion particles into the cell and plays an important role in inducing protective immunity against SARS-CoV-2^1^. The most notable therapeutic success in COVID-19 has been the development of monoclonal antibodies (mAbs) targeting the SARS-CoV-2 S protein^2^. S gene mutations are therefore of particular concern.

Numerous SARS-CoV-2 variants are circulating globally, but only a few are classified as variants of concern (VOCs) or variants of interest^3,4^. As of December 2021, five SARS-CoV-2 variants have been designated VOCs by the European Centre for Disease Control and Prevention due to their association with increased transmissibility, more severe disease, and reduced neutralisation by antibodies. These variants are B.1.1.7 (Alpha) (currently not a VOC), B.1.351 (Beta), P.1 (Gamma), B.1.617.2 (Delta), and the recently emerged B.1.1.529 (Omicron). SARS-CoV-2 variants exhibit varying susceptibility to mAbs, convalescent plasma, and plasma from vaccinated individuals^5^.

Monitoring variant surveillance is crucial to enable the implementation of countermeasures as early as possible^6^. The aim of this study was to show the evolution of SARS-CoV-2 variants in Madrid (Spain) during 2021 and the usefulness of reverse transcription-polymerase chain reaction (RT-PCR) testing for variant analysis in monitoring the pandemic.

## Methods

### Setting and design

This observational study was conducted at University Hospital 12 de Octubre (Madrid, Spain), which is a tertiary reference hospital for SARS-CoV-2 variant analysis. The study was carried out in accordance with the Declaration of Helsinki, as revised in 2013. Approval from a Research Ethics Committee was not required, as stated in Organic Law 3/2018, enacted on December 5, regarding the Data Protection and Guarantee of Digital Rights. This law allows health authorities and public institutions with public health monitoring powers to conduct scientific research without the consent of the data subject in situations of exceptional relevance and seriousness for public health.

In 2021, variant analysis of SARS-CoV-2 was conducted prospectively on the majority of initial positive samples, depending on the number of positive samples obtained during the peak waves and the availability of reagents. Variant screening was performed on samples collected from emergency departments, healthcare workers, hospitalizations, and three primary care centers designated as sentinel sites by local public health authorities, spanning a total of 22 weeks (1–11, 28–37, and 50–52). For the remaining 30 weeks (weeks 12–27 and 38–49), variant screening was conducted on all positive initial samples**.**

### Study procedures

The analysis of SARS-CoV-2 variants by RT-PCR was conducted using various diagnostic tests based on their availability and usefulness in detecting the emerging variants. The reagents used throughout the study are outlined in Table 1.

**Table 1:** RT-PCR and whole genome sequencing assays and RNA extraction methods used during the weeks of 2021 according to the emergence of new variants of concern and reagents availability.

Week	RT-PCR			Whole genome sequencing	
	Amplification reagents	Mutations detected	RNA extraction method	Reagents	RNA Extraction method
1–6	TaqPath COVID-19 assay (Thermo Fisher Scientific, Waltham, MA, USA)	H69-V70 deletion	EasyMag (bioMérieux Diagnostics, Marcy-l'Etoile, France)	Ion Torrent (Thermo Fisher Scientific, Waltham, MA, USA)	EasyMag (bioMérieux Diagnostics, Marcy-l'Etoile, France)
7–11	VirSNiP SARS-CoV-2 Spike N501Y, del H69/V70, E484K TIB Molbiol (Berlin, Germany)	H69-V70 deletion N501Y, E484K	EasyMag (bioMérieux Diagnostics, Marcy-l'Etoile, France)		
12–24	Allplex SARS-CoV-2 Variants I Assay (Seegene, Seoul, South Korea)^a^	H69-V70 deletion N501Y, E484K	Microlab STARlet (Seegene, Seoul, South Korea)	Ion Torrent (Thermo Fisher Scientific, Waltham, MA, USA) and ABL SA Group (Luxembourg/Illumina, San Diego, CA, USA)^b^	
25–32	VirSNiP SARS-CoV-2 Spike L452R TIB Molbiol (Berlin, Germany)	L452R	Microlab STARlet (Seegene, Seoul, South Korea)	ABL SA Group (Luxembourg/Illumina, San Diego, CA, USA)	
33–52	Allplex SARS-CoV-2 Variants II Assay (Seegene, Seoul, South Korea)	K417N, K417T, L452R, W152C	Microlab STARlet (Seegene, Seoul, South Korea)		

^a^Allplex SARS-CoV-2 Variants I was performed from week 12 to 52, along with VirSNiP SARS-CoV-2 Spike L452R (during weeks 25–32) and Allplex SARS-CoV-2 Variants II (during weeks 33–52).

^b^Ion Torrent or ABL platforms were used during 12–24 weeks according to the reagents availability.

The results were interpreted as follows: Alpha variant (S gene dropout or positive for N501Y and del H69/V70); Beta/Gamma (positive for N501Y and E484K); Delta (positive for L452R); and Omicron (positive for N501Y, del H69/V70 and K417N).

Whole genome sequencing (WGS), considered the gold standard technique, was performed on samples that had been previously analysed for variant using RT-PCR and had a Ct value < 25. The decision to perform WGS also depended on the number of positive samples obtained during peak waves and the availability of reagents. As result, WGS was conducted on 10.8% of the samples that underwent SARS-CoV-2 variant analysis by RT-PCR. Furthermore, when it was not feasible to perform WGS on all samples that had been previously analysed by RT-PCR (during peak waves), the selection of samples for WGS was based on random criteria. These selection process ensured representation from a diverse set of samples. WGS was carried out from the original sample with Ion Torrent (Thermo Fisher Scientific, Waltham, MA, USA) or with ABL SA Group (Luxembourg/Illumina, San Diego, CA, USA) platforms, depending on reagent availability (Table 1). Both methods followed the ARTIC nCoV-2019 sequencing protocol^7^. Specifically, for Ion Torrent platform, base calling, trimming and quality control were performed using the built-in pipeline in the Ion Reporter™ Software. The reads were then mapped against the reference genome Wuhan-Hu-1 (GenBank accession number: MN908947.3) using the IRMA assembler^8^ plugin of the Ion Reporter™Software (ThermoFisher Scientic, Carlsbad, CA, USA). Variant calling was carried out in parallel with the Variant Caller plugin of the Ion Reporter™ Software and with Snippy v.4.6.0^9^, with default settings (10 times minimum coverage and 90% minimum allele frequency). Annotations of variants were based on the reference genome. The Variant Caller plugin was also used to search for minor variants. To filter these, the reads were mapped to the reference strain using the Geneious Prime software (version 2020.0.4, Biomatters Ltd., New Zealand), and the variants were manually checked over the alignments. All the variants with low coverage, present in less than 15% of the reads, located near the ends of the reads (amplicons) or with low read qualities were discarded. Variants identified as problematic by De Maio et al.^10^ were also discarded. For the ABL platform, different stages of the pipeline were performed using the MicrobioChek software (ABL SA Group, Luxembourg)^11^. Lineages were assigned following the PANGO scheme^12^ and employing the Pangolin 2.0 web app^13^, and the NextStrain year-letter scheme on the NextClade web page^14^. All SARS-CoV-2 sequences were deposited in the GISAID database^15^ (code: h-CoV-19/Spain/MD-H12O).

### Statistical analysis

The qualitative variables are presented as numbers and percentages. The figures were created using GraphPad Prism, version 6.

## Results

**Figure 1:**
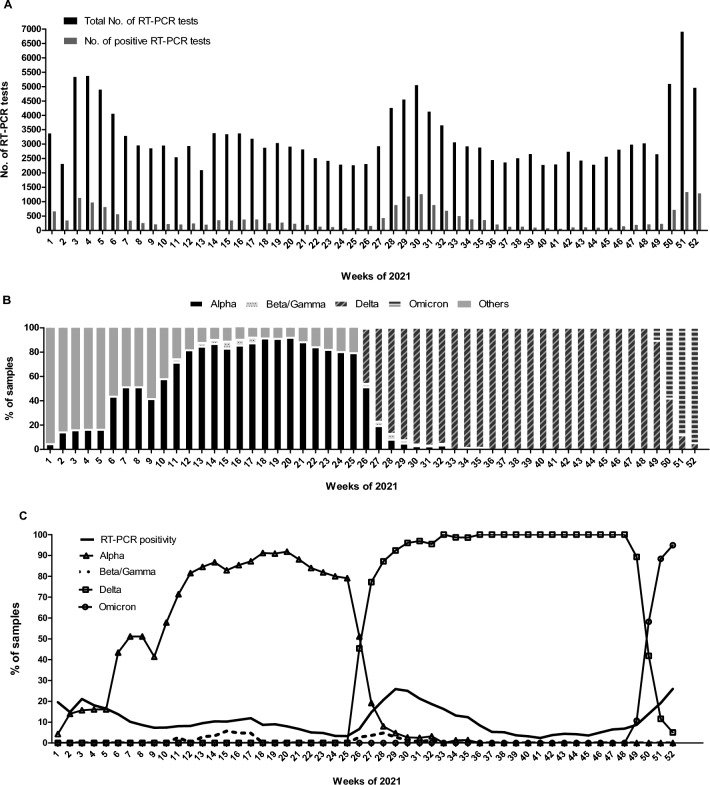
(**A**). Total number of nasopharyngeal samples tested for SARS-CoV-2 RT-PCR (initial and follow-up samples, n = 168,014) and total number of SARS-CoV-2 positive samples (initial and follow-up samples, n = 20,812) during 2021. (**B**) Distribution of SARS-CoV-2 variants in the first SARS-CoV-2 RT-PCR positive samples during 2021. (**C**) Percentage of positive SARS-CoV-2 RT-PCR samples (red) and percentage of SARS-CoV-2 variants per week.

In 2021, our laboratory analysed a total of 168,014 samples, which included diagnostic and follow-up samples. Among these, 20,812 samples (12,4%) tested positive for SARS-CoV-2 RNA. Variant analysis was performed on the first samples from 9315 individuals with a cycle threshold (Ct) value below 35. These samples were obtained from various sources: 16.2% from emergency departments, 17.2% from hospitalisations and 66.6% from primary care centres. The distribution of the tested samples by week is depicted in Fig. 1A. The Alpha variant was first detected around week 3; followed by a slight increase in Beta/Gamma variants was observed during week 15. The Delta variant emerged around week 29, and the Omicron variant appeared at week 50. The distribution of SARS-CoV-2 variants throughout 2021 is illustrated in Fig. 1 (panels B and C), along with the positivity rate of diagnostic RT-PCR (panel C). As shown in Fig. 1, the Delta and Omicron variants emerged explosively. Just three weeks after their appearance, they accounted for nearly all the cases. Regarding the Omicron variant, the first case was detected in our centre on December 7, 2021, and within five days (by December 12), it represented 30.0% of cases. The prevalence of Omicron continued to rise on a weekly basis, reaching 93.0% of cases by the end of December.

WGS was performed on 1002 samples, and there were no discrepancies between the WGS and RT-PCR results. The positive and negative predictive values with 95% confidence interval, for the mutations detected by each RT-PCR assay, using WGS as the reference method, were 100.0%. WGS helped to distinguish between the Beta and Gamma variants; 61 samples categorized as Beta/Gamma with the variant screening were differentiated as Beta (25/61, 41%) and Gamma (36/61 59%). WGS detected 196 cases of Alpha variant, 424 of Delta variant, 130 of Omicron variant (lineages: 80.8% BA.1.17, 13.1% BA.1.1.1, 3.8% BA.1, 1.5% BA.1.1 and 0.8% BA.1.15.1), 15 of Mu variant and 7 of Lambda variant. In addition, 21 samples presented the B.1.575 variant, while 148 samples belonged to other variants (54.1% B.1.177, 16.9% B.1, 6.8% B, 2.7% B.1.1, 2.7% B.1.525, 2.7% B.40, 1.4% B.1.153, 1.4% B.1.160, 1.4% B.1.258, 1.4% B.1.628, 0.7% B.1.1.222, 0.7% B1.1.28, 0.7% B.1.1.306, 0.7% B.1.1.318, 0.7% B.1.1.33, 0.7% B.1.1.519, 0.7% B.1.214.2, 0.7% B.1.526, 0.7% B.1.609, 0.7% B.1.625, 0.7% Q.1, 0.7% W.4 and 0.7% Z.1).

## Discussion

WGS is the gold standard for analysing mutations in the SARS-CoV-2 genome. However, it requires well-trained staff and specific facilities and is time-consuming and expensive^16^. Therefore, the number of samples that can undergo WGS is limited, and the results are usually obtained one week after the SARS-CoV-2 diagnosis. In contrast, variant analysis using RT-PCR is a simpler, less expensive and faster technique that can be quickly adapted to newly identified VOCs.

Constantly monitoring the pandemic is crucial^4,17^ because knowledge of SARS-CoV-2 variants is useful for selecting the treatment (the susceptibility of mAbs varies depending on the specific lineages within the SARS-CoV-2 Omicron variant)^18^ and for monitoring outbreaks and the emergence of sequential mutations in persistent cases of COVID-19^19^. The pandemic evolves rapidly, and its incidence increases with each new variant that arises. It is essential to analyse the variants on a daily basis and in as many positive samples as possible, to select the most appropriate therapy for individual patients and to promptly investigate epidemic clusters. The only way to achieve this goal is by performing variant analysis by RT-PCR. The Allplex Variant Assays I and II have proven to be highly useful for this purpose, as previously reported^20^. The Variant I Assay allows for simultaneous detection of the virus through RNA-dependent RNA polymerase amplification, providing a Ct value, which is crucial for the applicability of the WGS technique. In our experience, samples with a Ct < 35 show reliable results for RT-PCR variant screening, while a Ct < 25 is preferred for WGS analysis.

In this study, there was a complete concordance between RT-PCR and WGS results. RT-PCR can serve as a valuable tool in the identification of samples lacking the prevailing key mutations patterns, potentially indicating the emergence of a novel VOC. In such instances, WGS is necessary to definitively confirm the presence of the variant and to gain comprehensive insights into its genetic composition. Furthermore, the continuous monitoring of SARS-CoV-2 mutations using RT-PCR can be valuable in identifying the emergence of mAbs resistance subsequent to mAbs treatment or prophylaxis. Nevertheless, it will always be essential to sequence some samples with a pattern that is clearly associated to a specific variant in order to monitor the appearance of new mutations. In addition, Allplex variants assays only detect mutations in spike gene, while WGS can detect mutations throughout the entire genome. As SARS-CoV-2 continues to diversify, it may become increasingly challenging to distinguish between numerous sublineages using RT-PCR. For example, current RT-PCR methods cannot differentiate between sublineages of the Omicron variant.

This study has certain limitations, such as nor performing SARS-CoV-2 variant analysis on all initial positive samples; however, the sample size was large. The reagents for variant analysis changed during the study due to fluctuating availability during the pandemic; nevertheless, Seegene reagents were the most often used. Additionally, we could not distinguish between the Beta and Gamma variants due to the unavailability of the Allplex SARS-CoV-2 Variants II Assay at the time of detection.

This study presented a large series of SARS-CoV-2 variant analysis, highlighting the usefulness and reliability of variant screening by RT-PCR to follow the day-by-day evolution of the SARS-CoV-2 pandemic.

In conclusion, continuous surveillance of SARS-CoV-2 variants is essential, and RT-PCR is a highly useful method, specially during periods of high COVID-19 incidence. RT-PCR is a technique that can be implemented in all SARS-CoV-2 diagnostic laboratories. However, WGS remains necessary for the detection of all SARS-CoV-2 variants and lineages, as well as for monitoring mutations of the spike gene.

## Data Availability

The datasets generated during and/or analysed during the current study are available from the corresponding author on reasonable request.
